# Axial Compressive Behavior of Cross-Shaped CFST Stub Columns with Steel Bar Truss Stiffening

**DOI:** 10.3390/ma16114147

**Published:** 2023-06-02

**Authors:** Yu Tao, Chao Gong, Sumei Zhang, Xiaozhong Li, Xiao Tan, Junjie Hu

**Affiliations:** 1 School of Civil and Environmental Engineering, Harbin Institute of Technology, Shenzhen, University Town, Shenzhen 518055, China; 2 Central Research Institute of Building and Construction Co., Ltd., MCC, No. 33, Xitucheng Road, Beijing 100088, China

**Keywords:** cross-shaped CFST, steel bar truss stiffening, axial compression behavior, bearing capacity, ductility

## Abstract

Concrete-filled steel tube (CFST) columns have been widely used in residential buildings due to their high bearing capacity, good ductility, and reliable seismic performance. However, conventional circular, square, or rectangular CFST columns may protrude from the adjacent walls, resulting in inconvenience in terms of the arrangement of furniture in a room. In order to solve the problem, special-shaped CFST columns, such as cross-shaped, L-shaped, and T-shaped columns, have been suggested and adopted in engineering practice. These special-shaped CFST columns have limbs with the same width as the adjacent walls. However, compared with conventional CFST columns, the special-shaped steel tube provides weaker confinement to the infilled concrete under axial compressive load, especially at concave corners. The parting at concave corners is the key factor affecting the bearing capacity and ductility of the members. Therefore, a cross-shaped CFST column with steel bar truss stiffening is suggested. In this paper, 12 cross-shaped CFST stub columns were designed and tested under axial compression loading. The effects of steel bar truss node spacing and column–steel ratio on the failure mode, bearing capacity, and ductility were discussed in detail. The results indicate that the columns with steel bar truss stiffening can change the final deformation mode of the steel plate from single-wave buckling to multiple-wave buckling, and the failure modes of columns also subsequently change from single-section concrete crushing failure to multiple-section concrete crushing failure. The steel bar truss stiffening shows no obvious effect on the axial bearing capacity of the member but significantly improves the ductility. The columns with a steel bar truss node spacing of 140 mm can only increase the bearing capacity by 6.8% while nearly doubling the ductility coefficient from 2.31 to 4.40. The experimental results are compared with those of six design codes worldwide. The results show that the Eurocode 4 (2004) and the Chinese code CECS159-2018 can be safely used to predict the axial bearing capacity of cross-shaped CFST stub columns with steel bar truss stiffening.

## 1. Introduction

Concrete-filled steel tube (CFST) columns are composite columns formed by filling concrete into steel tubes. The structure can fully utilize the material properties of steel tubes and concrete. Steel tubes provide high tensile strength and stiffness, while concrete offers high compressive strength and durability. Additionally, the presence of concrete helps to delay or prevent the premature local buckling of the steel tubes, thereby enhancing their local stability and maximizing their yield strength. Furthermore, external steel tubes provide lateral confinement to the concrete, resulting in triaxial compression and improving the strength, ductility, and toughness of the concrete. Due to their high bearing capacity, excellent ductility, and reliable seismic performance [1,2], CFST columns have been widely employed in practical engineering, particularly in high-rise residential buildings. However, conventional circular, square, or rectangular CFST columns can no longer meet the functional requirements of residential buildings. Thus, special-shaped CFST columns with better spatial layouts, such as cross-shaped, L-shaped, and T-shaped columns, have been suggested and adopted. The columns have limbs with the same width as the adjacent walls, avoiding column flanges protruding from the wall and thereby satisfying the requirements in terms of building aesthetics and furniture layout, improving the project’s usability and economic benefits [3]. However, compared to conventional section CFST columns, irregular-shaped steel tubes provide weaker confinement to the core concrete, which does not fully utilize the material properties and results in reduced bearing capacity and ductility of the members. Taking cross-shaped CFST columns as an example, under axial compressive load, both steel tubes and concrete experience compressive expansion, causing the outward displacement of the steel plates at the concave corners. As the lateral expansion of the concrete significantly increases, the steel plates near the concave corners tend to separate from the surface of the core concrete. Ultimately, the steel tube and concrete bear axial compressive loads separately, which fails to effectively enhance the axial compressive behavior of both components. Thus, the parting at the concave corners is the key factor affecting the bearing capacity and ductility of the members.

In order to solve the problem, scholars have adopted some appropriate measures to improve the bearing capacity or ductility of ordinary special-shaped CFST columns. Previous research has shown that the ductility of members can be improved to a certain extent by setting sawtooth reinforcement or butt-tensioning reinforcement at the concave corners or middle sides of the steel tubes [4,5,6,7]. Additionally, Cai Jian, Zuo Zhiliang et al. [8,9] proposed restraining the out-of-plane deformation of the steel tube by drilling holes in the outer wall of the steel tube and setting binding bars. Additionally, some scholars [10,11,12,13,14,15] have used vertical steel plates, angled steel, or open-hole steel plates to strengthen the bending rigidity of the steel plate section in order to achieve the common working effect of steel tubes and core concrete. Han Linhai [16], Tao Zhong [17], Cheng Rui [18], Tu Yongqing [19,20], and Zheng Yongqian [21] adopted a multi-cell form to eliminate the adverse effects of the concave corners of special-shaped CFST columns. The measures can improve the bearing capacity and ductility of special-shaped steel tube concrete columns to some extent, but there are also some issues, such as the complexity of the processing technology, the uneven side wall of the steel tube, and the insufficient improvement of the component’s ductility.

**Figure 1:**
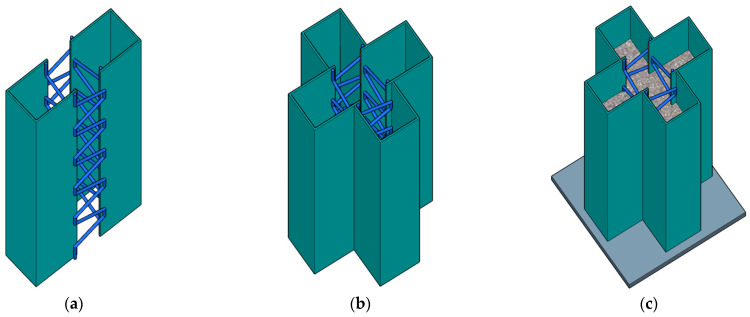
Cross-shaped CFST column with steel bar truss stiffening. (**a**) Steel bar truss welding schematic. (**b**) Assembly of grooved steel plates. (**c**) Closure plate welding and concrete pouring.

To address these issues, this paper proposes a novel type of cross-shaped CFST column with steel bar truss stiffening. The column is primarily composed of cold-formed grooved steel plates, bent steel bars, and core concrete. The manufacturing process involves welding bent steel bars to the edges of grooved steel plates (Figure 1a), then using two grooved steel plates to close-weld with the previous ones (Figure 1b), and finally completing production by pouring concrete and welding the closure plate (Figure 1c). Alternating welding of bent steel bars on inner steel plates at concave corners is expected to have several benefits. Firstly, it avoids multiple welding at concave corners of steel tubes, which reduces the risk of brittle fractures and structural damage caused by drilling on side walls or complex plug welding problems. Secondly, it avoids the protrusion of nuts on the side wall of the member, making it flatter and more aesthetic. Thirdly, oblique steel bars provide effective lateral tension for steel plates at concave corners, which restricts the out-of-plane displacement of steel tubes and enhances the confinement effect on concrete. Based on existing technology, profiled steel plates and steel trusses can be automated for bending and welding, enabling modular assembly. Furthermore, the proposed steel bar truss stiffening method used in this study involves utilizing a lower steel ratio of steel bars to significantly enhance the poor ductility of the members, leading to material cost savings. To promote the application of members in high-rise residential buildings, its basic axial compressive mechanical properties need studying. Further research should be conducted based on this study to investigate eccentric compressive behavior and seismic performance.

The paper aims to investigate the influence of steel bar truss stiffening on the mechanical characteristics of cross-shaped CFST stub columns under axial compression load. The failure mode, bearing capacity, ductility, and axial compression mechanisms of the members were investigated through axial compression testing on 12 short columns. The effect of steel bar truss node spacing and the steel ratio of steel tubes on the working mechanisms of concrete, steel tubes, and steel bars were analyzed. Based on the experimental results, some suggestions regarding the calculation method for the bearing capacity of cross-shaped CFST stub columns with steel bar truss stiffening have been proposed.

## 2. Test Program

### 2.1. Details of Specimens

**Figure 2:**
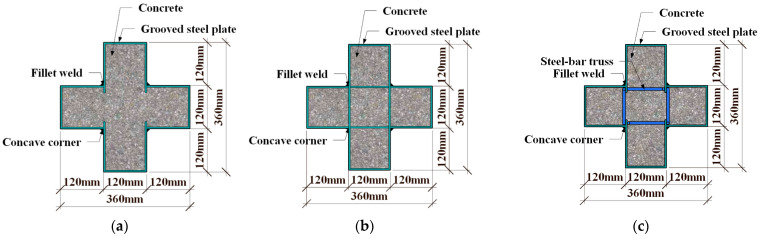
Cross-section forms of specimens. (**a**) unstiffened (Specimen C0). (**b**) multi-cell (Specimen CM6). (**c**) steel bar truss stiffening (other specimens).

**Figure 3:**
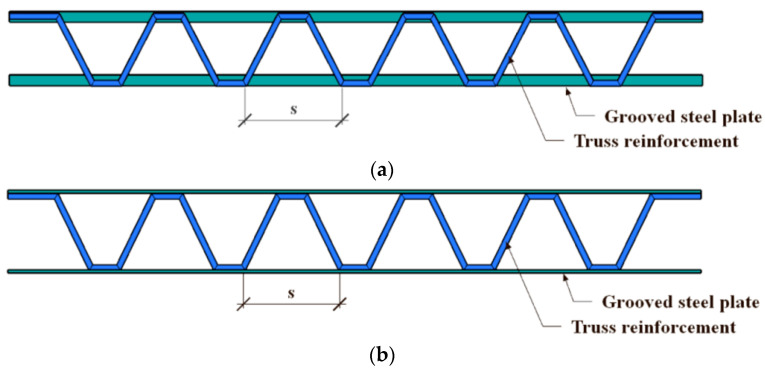
Diagram of the welding of steel bar truss. (**a**) Welded forms of steel bar truss I (A), (**b**) Welded forms of steel bar truss II (B).

In this study, a total of 12 specimens were designed and fabricated. The diameter of the steel bars inside the specimens is 8 mm. A limb-height-to-limb-thickness ratio of 1 was taken for the specimens, with both the limb height and limb thickness being 120 mm, which was calculated based on the thickness of walls of 240 mm, which are commonly used in residential buildings according to 1/2 scaling. In addition, in order to study the axial compressive behavior of the stub columns proposed in this paper, the height of the specimen is 1000 mm. The primary parameters considered in the test include cross-section forms of specimens (Figure 2), the steel bar truss node spacing (*s* = 140 mm, 200 mm, or 280 mm) (Figure 3) and the thickness of the steel tube (*t* = 3.50 mm or 5.35 mm). As shown in Table 1, *α* is the column–steel ratio, including two parts. One part is from the outer steel tube, which is called the steel ratio of the steel tube, *α*_1_, and the other part is from the steel bar truss, which is called the steel ratio of the steel bar, *α*_2_. *SI* is the strength index [22], which can be calculated in Equation (1), *N*_0_ is the superimposed bearing capacity of each material without considering the combined effect (Equation (2), *N*_u_ represents the measured value of the peak load of the specimen. *DI* is the ductility coefficient [23], which can be calculated as in Equation (3). *ε*_0.75_ is the average longitudinal strain when the load is increased to 75% of the peak load. *ε*_0.85_ is the average longitudinal strain when the load is reduced to 85% of the peak load.

*SI* = *N*_u_/*N*_0_ (1)

*N*_0_ = *f*_c_
*A*_c_ + *f*_y_
*A*_s_ (2)

*DI* = *ε*_0.85_/*ε*_0.75_ (3)

### 2.2. Material Properties

C50 strength grade commercial concrete was poured on-site, and the material compression tests were carried out according to the Chinese standard GB/T50081-2019 [24]. The compressive strength of the concrete, the modulus of elasticity, and Poisson’s ratio were measured using 4 reserved prismatic test blocks (150 mm × 150 mm × 300 mm). After testing, the average compressive strength *f*_c_ is 46.6 MPa, the modulus of elasticity *E*_c_ is 31.6 GPa, and Poisson’s ratio *μ*_c_ is 0.237.

The steel tubes were made of Q235 grade steel, and the steel bar trusses were made of bright round steel with a higher strength grade, and the material tensile tests of the steel tubes and steel bars were carried out in accordance with the Chinese standard GB/T 228–2010 [25]. The measured mechanical property indexes of the steel are shown in Table 2, where *f*_y_ is the yield strength, *f*_u_ is the ultimate strength, and *E*_s_ and *μ*_s_ are the elastic modulus and Poisson’s ratio, respectively.

### 2.3. Test Set-Up and Measurement

**Figure 4:**
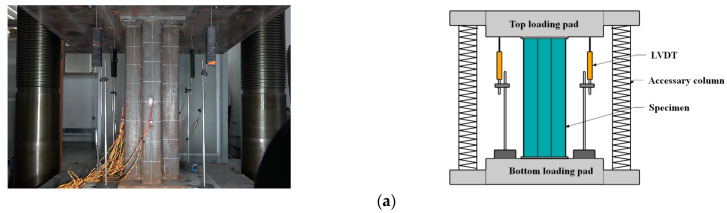

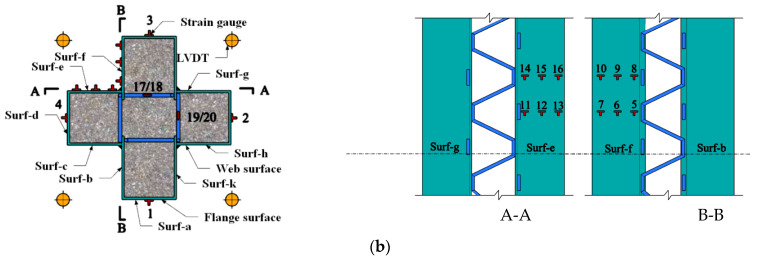
Test set-up and measuring equipment used in axial loaded test. (**a**) Test set-up. (**b**) Measuring equipment.

A 30,000 kN universal testing machine (Figure 4a) was employed to carry out the axial compressive test. Before formal loading, the specimen was geometrically and physically aligned, and force control was utilized with a constant rate of 1 kN/s until the predicted peak load was reached. Following this, displacement control was implemented with a rate of 0.3 mm/min until failure occurred. The load data were measured using the built-in force transducer of the testing machine, while the displacement data were collected via 4 linear variable displacement transducers (LVDTs), which were employed to capture the average axial deformation between the two end caps. The strain data were collected by 20 groups of strain gauges, with 4 groups positioned on the flange surface of the steel tube, 12 groups of lateral and longitudinal strain gauges glued onto the web surface of the steel tubes, and the remaining 4 strain gauges glued onto the steel bars, which measured the axial strains along the oblique steel bars near the mid-height of the specimens. For specimen C0 and specimen CM6, the arrangement of the strain measuring points of the outer steel tube is consistent with that of the specimen with steel bar truss stiffening. The measuring points of the specimens with steel bar truss stiffening are depicted in Figure 4b.

## 3. Test Results and Discussion

### 3.1. Test Phenomena and Failure Modes

**Figure 5:**
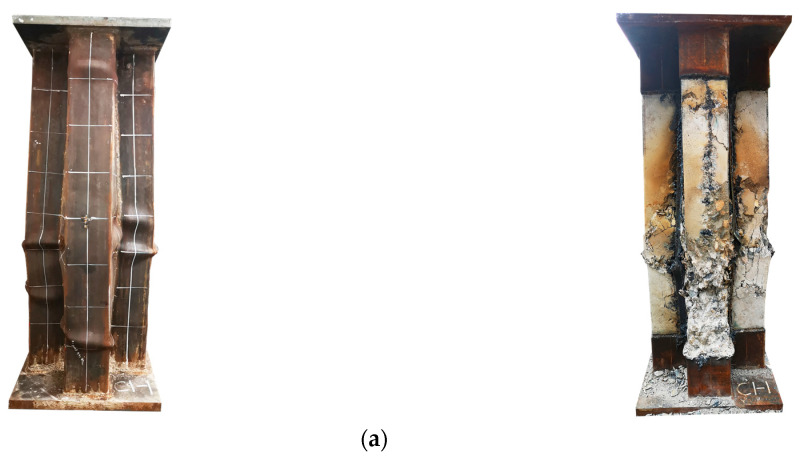

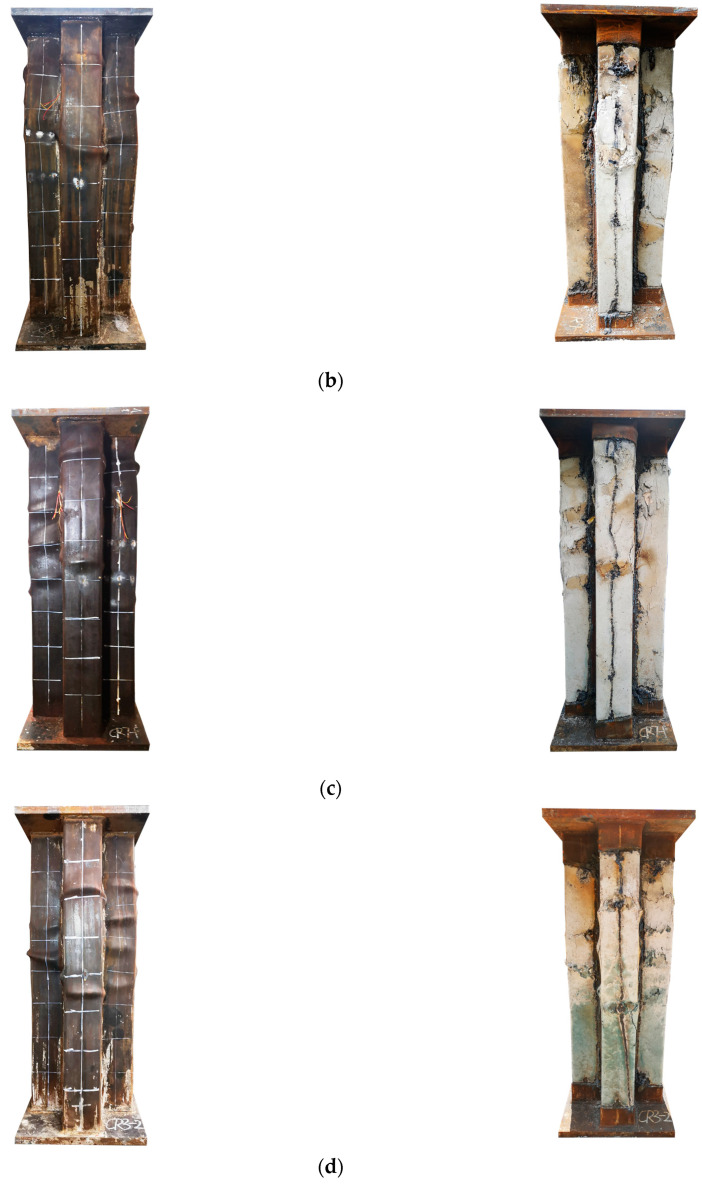
Failure patterns of specimens. (**a**) C0, (**b**) CR1-1, (**c**) CR2-1, (**d**) CR3-2.

For all specimens, only slight overall out-of-plane deformation of the steel tube could be observed before reaching the peak load. However, after reaching the peak load, the failure mode of the steel tubes with different stiffening forms changed and can be categorized into two typical modes. The first mode is characterized by the bulging failure of the specimen, which occurs due to the separation between the steel plates at the concave corner from the concrete near the mid-height of the column. The second mode is the local bulging failure of the specimen within a certain height range, with no significant lateral deformation observed. The typical failure modes of the specimens are illustrated in Figure 5.

Regarding the unstiffened specimen C0 (Figure 5a), when the specimen was damaged, the specimen exhibited a single-wave buckling deformation along the web surface of the steel tube in the height direction of the column, with maximum lateral deformation occurring near the mid-height of the specimen. Additionally, a cracking sound was audible from the local concrete cracking. The loading was stopped when the load dropped to 1391 kN (0.30 *N*_u_), at which point the concave corner of the steel tube located in the mid-height of the specimen had undergone a change from 90 degrees to approximately 100 degrees. After cutting open the outer steel tube of specimen C0, it was discovered that the steel tube separated from the concrete at the concave corner. Furthermore, the concrete at the concave corners had developed cracks along the height of the column. The crack’s width progressively widened from both ends to the column’s middle section, peaking at approximately 20 mm. In the middle section, the concrete close to the concave corner exhibited obvious crushing. In contrast, the concrete in the column limb, situated farther from the concave corner, experienced lighter crushing. The main reason is that after the peak load is reached, the expansion of the concrete in each column limb intensifies, while the lateral confinement of the steel plates on the core concrete at the far end of the section in the column is weaker, resulting in a gradual increase in the distance of the concrete in each column limb from the geometric center of the section, which leads to a certain eccentric compression effect on the concrete in each column limb. As a consequence, the steel plates on both sides of the concave corner detached from the core concrete, resulting in the steel tube’s inability to provide effective confinement to the core concrete.

For specimens with steel bar truss stiffening, as the steel bar truss node spacing decreases from 280 mm to 140 mm, the failure mode gradually changed. Specifically, specimen CR1-1, with a node spacing of 280 mm, still exhibited mainly drum-shaped damage (Figure 5b). The column end face showed a single-wave bulge along the column height direction, with a smaller radius than that of the unstiffened specimen. The maximum bulge was concentrated near the section, 150 mm from the mid-height of the column. Upon cutting open the steel tube of specimen CR1-1, the internal concrete in the bulging part of the steel tube was found to have been crushed, and the lateral concrete crushing was relatively severe compared to the end face of the column limb. When the node spacing was reduced to 200 mm, the overall convexity of specimen CR2-1 (*s* = 200 mm) was not apparent (Figure 5c), a multi-sectional local bulge phenomenon was observed on the surface of the steel tube, and the concave corner of the steel tube was better restricted, not showing a more obvious out-of-plane displacement. After cutting open the steel tube of the specimen, the concrete of the column limb exhibited multiple instances of crush damage. Compared with specimen CR1-1, there were more crushed locations on the side of the column, with a wider distribution range arranged at certain intervals along the height direction of the column. With a further reduction in the steel bar truss node spacing, specimen CR3-2 (*s* = 140 mm) still exhibited the failure mode of multi-wave bulging on each side (Figure 5d), and the extension range of the wave tended to increase, while the local steel tube bulging appeared at the end of the column limb without obvious overall convexity. Moreover, according to the steel tube deformation, it could be observed that the two lateral steel plates (web surfaces) adjacent to the concave corner showed a staggered distribution of crests or troughs. After cutting open the external steel tube, the lateral edges of the adjacent column limbs showed a staggered arrangement distribution of crush points, consistent with the bulge wave distribution of the steel tube. The deformation pattern of the outer surface of the member is related to the location and steel bar truss node spacing. The smaller the steel bar truss node spacing, the closer the arrangement of the concrete crushing points and the more uniform the longitudinal stress distribution of the core concrete section. The stress concentration level is thus lower, which enhances the overall deformation performance of the member.

In summary, for traditional cross-shaped CFST stub columns, the steel tube near the concave corner is prone to separate from the concrete, which results in the ineffective confinement of the core concrete by the steel tube during the later stage of loading. Consequently, the concrete near the mid-height of the column is crushed, resulting in a waist-drum-shaped failure mode. However, this failure mode can be altered by welding steel bars to the inner side of the steel tube at the concave corners. When the steel bar truss node spacing is reduced to a certain range, the oblique steel bars spaced along the high direction of the column provide effective lateral restraint for the steel tube, preventing concrete cracking at the concave corners and the out-of-plane displacement of the steel plates. As a result, the lateral confinement effect of the steel tube on the core concrete is enhanced.

### 3.2. Test Set-Up and Measurement

The experimental results shown in Section 3.1 demonstrate that steel bar truss stiffening can enhance the ultimate bearing capacity and deformation capacity of the members. However, the degree of improvement may vary due to the different steel bar truss node spacings. Therefore, the next step of the study is to compare and analyze the impact of different node spacing on the axial compressive behavior of cross-shaped CFST stub columns.

**Figure 6:**
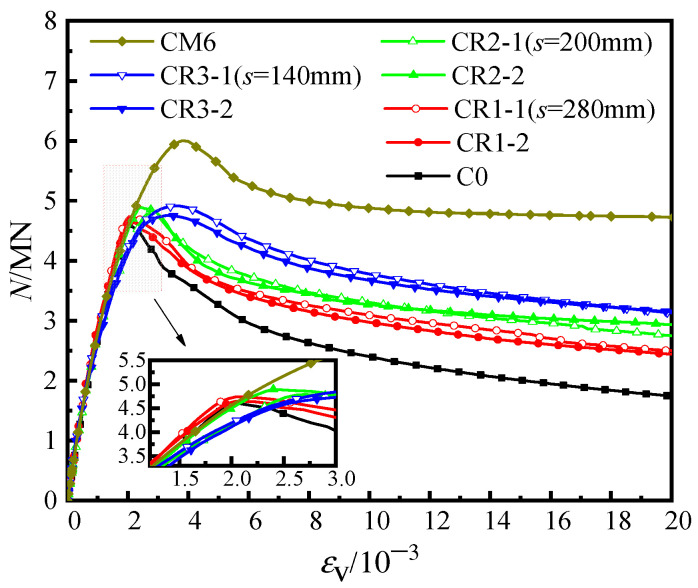
Load-averaged longitudinal strain curves of the specimens.

**Figure 7:**
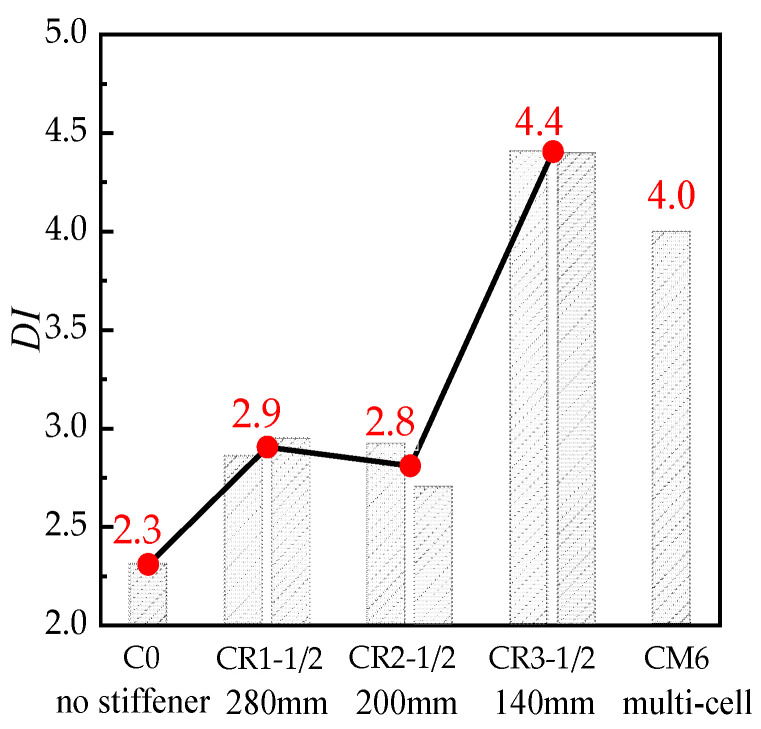
Bar chart of the ductility of the specimens.

Figure 6 shows the load-averaged longitudinal strain (*N-ε*_v_) curves of the unstiffened specimen, C0, the multi-cell specimen, CM6 and three groups of specimens with steel bar truss stiffening (*s* = 140 mm, 200 mm, or 280 mm). In the early loading stage, the initial stiffness of all specimens was similar (Figure 6). As the specimens entered the elastic–plastic stage, there were slight variations in the curves due to the discreteness of concrete pouring quality, which had a minimal influence on the ultimate bearing capacity and deformation ability of the concrete during the loading process. When the peak load was reached, the axial bearing capacity of the specimens with node spacings of 280 mm (CR1-1/2), 200 mm (CR2-1/2), and 140 mm (CR3-1/2) increased by 0.9~3.0%, 3.9~6.3%, and 3.3~6.8%, respectively, compared with the unstiffened specimen C0. Continuing to load until the specimens were destroyed, the load of the unstiffened specimen, C0, dropped faster and had poor ductility. It can also be seen in Figure 5a from Section 3.1 that the steel tube deformation at the mid-section of specimen C0 was large, the inner concrete was severely crushed, and the ductility was poor. For specimens with steel bar truss stiffening, as the steel bar truss node spacing was reduced from 280 mm to 140 mm, the curve tended to be gentle, and the ductility of the specimen was improved. In contrast to the multi-cell specimen, CM6, the bearing capacity of the specimens with steel bar truss stiffening was lower. The main reason is that the steel ratio of the multi-cell specimen was 10.29%, which was significantly higher than that of the specimens with a steel bar truss (about 8.81%). To evaluate the ductility of various specimens, a bar chart of their ductility coefficients was plotted and compared (Figure 7). Compared with the unstiffened specimen, C0, the ductility coefficients of specimens with steel bar truss node spacings of 280 mm (CR1-1/2), 200 mm (CR2-1/2), and 140 mm (CR3-1/2) were increased by 23.9~27.6%, 17.0~26.4%, and 90.3~90.9%, respectively. It indicates that the ductility of the specimens can be significantly improved when the steel bar truss node spacing is reduced to 140 mm. Additionally, compared to the multi-cell specimen, the ductility coefficients of the specimens with a node spacing of 140 mm are close to the multi-cell specimen, indicating that the members with a reasonable setting of steel bar truss node spacing can achieve the same vertical deformation capacity as the multi-cell member.

**Figure 8:**
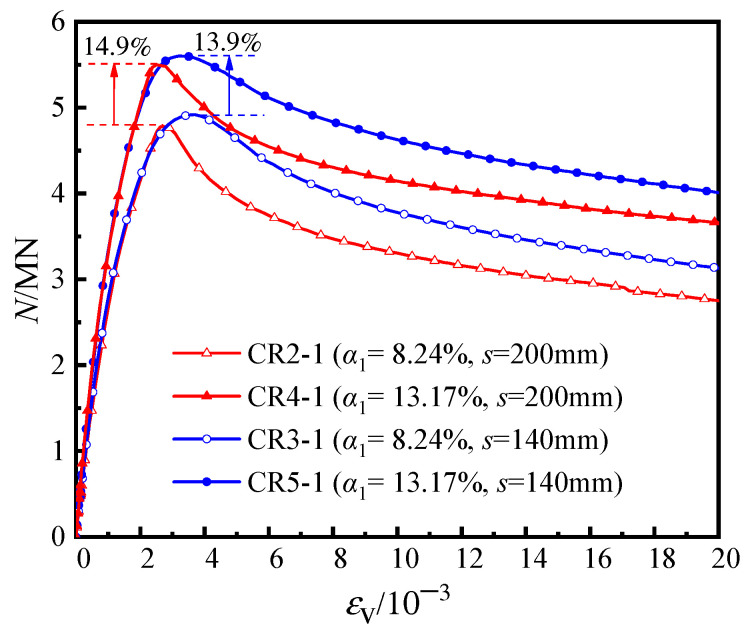
Load–averaged longitudinal strain curves of the specimens.

Figure 8 displays the load-averaged longitudinal strain (*N*-*ε*_v_) curves of the specimens with different steel ratios. When the steel ratio of the steel tube increased from 8.24% to 13.17%, the elastic stiffness improved correspondingly. Moreover, the axial bearing capacity of specimens CR5-1 (*α*_1_ = 13.17%; *s* = 140 mm) and CR4-1 (*α*_1_ = 13.17%; *s* = 200 mm) increased by 13.9% and 14.9%, respectively, and the ductility coefficient also increased by 37.7% and 28.4%, respectively. The results show that the increase in the bearing capacity and ductility coefficients of the specimens with different node spacing are similar, indicating that for members with a certain range of steel bar truss node spacing (*s* = 140 mm to 200 mm), increasing the steel ratio of the steel tube has a similar effect on the bearing capacity and ductility of the members. Furthermore, compared with specimen CR4-1 (α = 13.17%; *s* = 200 mm), the increase in the bearing capacity of specimen CR5-1 (α1 = 13.17%; *s* = 140 mm) was minimal (approximately 0.08%), while the improvement in the ductility coefficient was significant, increasing from 3.75 to 6.03.

## 4. Test Results and Discussion

### 4.1. Stress Development of Steel Bars in Truss

**Figure 9:**
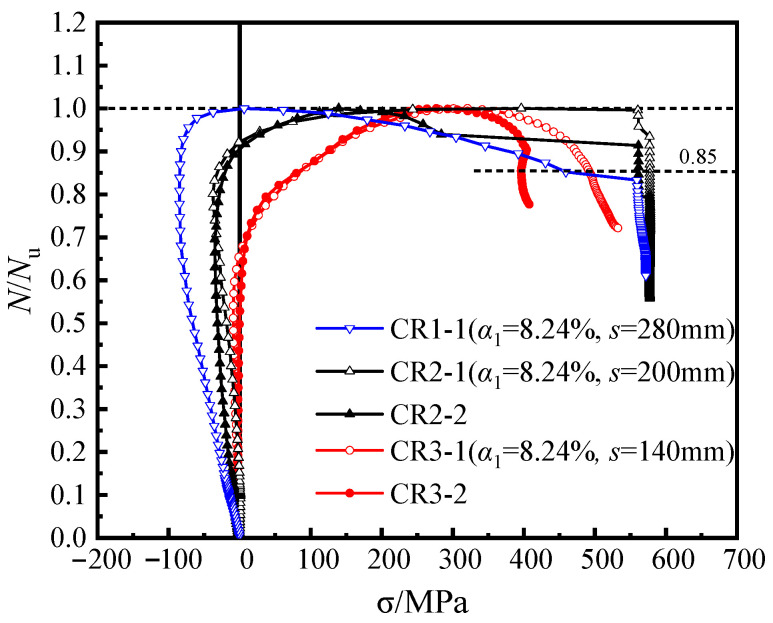
Stress curves of steel bars (*α*_1_ = 8.24%).

To directly evaluate the out-of-plane restraining effect of the reinforcement in the truss on the steel plates at the concave corners during axial compression loading, the average stresses of the diagonal steel bars near the mid-height of the column (Figure 9) were measured and analyzed.

The stress development of steel bars in the truss undergoes three distinct processes: linear compression, nonlinear compression, and tension in all specimens. Specifically, at the initial stage of loading, there is minimal interaction between the steel tube and the core concrete. The steel tube and the core concrete are subjected to axial compression load alone, and the stresses in steel bars grew linearly in all specimens. As the axial load increased, the lateral expansion of the core concrete gradually exceeded that of the steel tube. Consequently, parts of the steel plates were pulled away from the core concrete, causing separations between the steel tubes and concrete. For specimen CR1–1, with a node spacing of 280 mm (Table 3), as the load increased to 4120 kN (0.87*N*_u_), the compressive stress reached a maximum value of 83.7 MPa. Thereafter, the lateral deformation of the concrete continued to increase, and when loaded near the peak load of 4740 kN (*N*_u_), the steel bars started to be subjected to tensile forces. As the loading continued to 4039 kN (0.85*N*_u_), the reinforcement tensile stress increased rapidly to 459.1 MPa. This indicates that before reaching the peak load, the steel bars basically did not play a role in restricting the out-of-plane displacement of the steel tube at the concave corner, while it is only after reaching the peak load, when the lateral deformation of the specimen is large, that the steel bars acted as a better tensile force.

To study the influence of steel bar truss node spacing on the mechanical behavior of the specimens, the normalized load (*N*/*N*_u_)—the steel bar stress (*σ*) curves of different specimens with steel bar truss stiffening (Figure 9)—were compared and analyzed.

When the node spacing was reduced, the maximum compressive stress of the steel bar grew smaller, and the steel bars started to play a restraining role on the steel plates earlier before reaching the peak load (Figure 9). The tensile stress increased rapidly with the continuous loading, and by comparing the axial deformations of the specimens, it can be found that the smaller the steel bar truss node spacing, the better the axial compression deformation capacity. Typical specimen CR2-1 (*s* = 200 mm) and specimen CR3-1 (*s* = 140 mm) are taken as examples (Table 3). When the axial load of the two specimens reached 0.92 *N*_u_ and 0.65 *N*_u_, respectively, the steel bars started to change from the compressive stress state to the tensile stress state, at which time the steel bars started to restrict the out-of-plane deformation of the steel plate at the concave corner. When the load came close to its peak, the tensile stresses of the steel bars in the truss reached 396.3 MPa and 320.8 MPa, respectively, and the steel bars did not yield. When the specimen was loaded until it was damaged, the tensile stress of the steel bars reached 577.3 MPa and 492.7 MPa for the specimens with 200 mm and 140 mm node spacing, respectively; therefore, the tensile effect of the steel bars was given full play. Especially for specimen CR3-1 and specimen CR3-2, with node spacings of 140 mm, immediately after the specimen entered the elastic-plastic stage, the steel bars played an out-of-plane restraining role on the steel tube at the concave corners.

**Figure 10:**
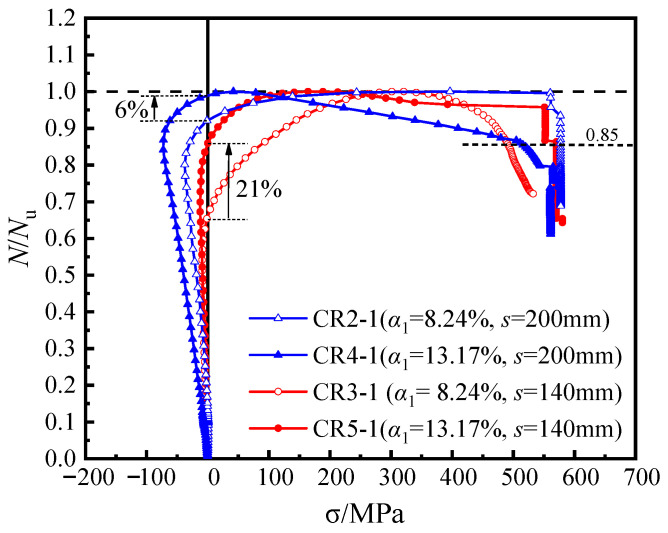
Stress curves of steel bars (*α*_1_ = 13.17%).

With the increased steel ratio of the steel tube employed, the tensile effect of the steel bar on the steel tube at the concave corner was delayed. This is evidenced by the increased normalized load of specimen CR5-1 (*α*_1_ = 13.17%; *s* = 140 mm) compared to specimen CR3-1 (*α*_1_ = 8.24%, *s* = 140 mm) (Table 3), which increased from 0.65 to 0.86 (Figure 10). Similar development patterns were observed in specimens CR2-1 (*α*_1_ = 13.17%; *s* = 200 mm) and CR4-1 (*α*_1_ = 13.17%; *s* = 200 mm), indicating that an increased steel ratio improved the bending stiffness of the steel plate and enhanced the confinement effect on the core concrete to some extent. When the lateral deformation of the core concrete intensifies and overcomes the lateral resistance of the steel tube, the tensile stress of the steel bars rapidly increases and begins to restrict the out-of-plane deformation of the steel plates at the concave corners, thereby improving the confinement of the steel tube to the concrete. Consequently, the confinement effect of the cross-shaped steel tube on the core concrete occurs in two stages: the constraint effect of the bending stiffness of the steel plate section on the core concrete during the first stage and the restraining effect of the steel tube at the concave corner by the internal steel bars of the specimen during the second stage. However, in the first stage, the confinement effect of the steel tube on the concrete is relatively small due to the irregular cross-section of the cross-shaped steel tube. Therefore, the increased steel ratio of the steel tube in this study does not significantly enhance the deformation capacity of the core concrete.

In summary, the smaller the steel bar truss node spacing, the timelier and more effectively the steel bars restrict the steel tube, and the better the steel tube confines the concrete within a certain range of node spacing between 140 mm and 280 mm. When the node spacing is 140 mm, the steel bars already exhibit tensile effects as soon as the specimen enters the elastic–plastic stage, which enhances the bearing capacity. However, the effect is not very noticeable. On the other hand, the axial deformation capacity of the member during the later stage of loading is significantly improved. As the steel ratio of the steel tube increases, the tensile effect of steel bars on the member remains noticeable, particularly after reaching the peak load, where it remains the primary factor in enhancing the confinement effect of the steel tube on the concrete.

### 4.2. Stress Development of Steel Tube

**Figure 11:**
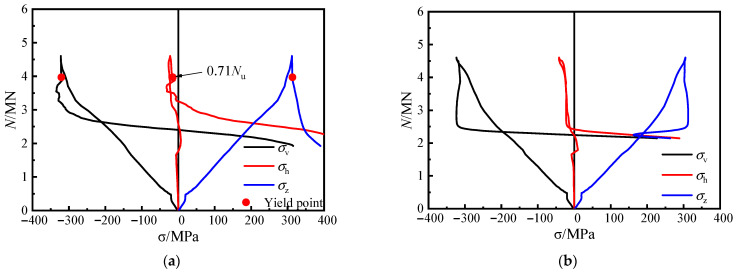
Load–stress curves of medium section steel tubes of specimen C0. (**a**) 5#, (**b**) 6#.

**Figure 12:**
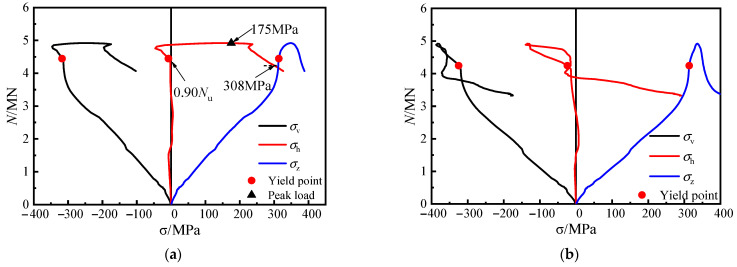
Load–stress curves of medium section steel tubes of specimen CR3-1. (**a**) 5#, (**b**) 6#.

To further illustrate the confinement effect of steel bar truss stiffening on the steel plates at the web surfaces of the specimen, the strain on the web surfaces was measured according to the measurement scheme (Figure 4b). The longitudinal stress (*σ*_v_), the lateral stress (*σ*_h_) and the equivalent stress (*σ*_z_) at each measuring point of the steel tube were obtained via the elastic–plastic stress analysis method [26]. The load–stress curves for the unstiffened specimen and the specimens with steel bar truss stiffening are shown in Figure 11 and Figure 12. Positive values indicate tension, while negative values indicate compression.

Through the stress curves of the steel plates at measuring points 5 and 6 of the unstiffened specimen C0, it can be seen that before reaching the peak load, the steel plates did not yield. The steel plates were mainly subjected to longitudinal loading, while the lateral stress developed slowly. After the peak load, the longitudinal stress curves of the two measuring points exhibited a rapid decrease in load while the stress value essentially remained constant. This indicates that the increased lateral deformation of the concrete caused the separation of the steel plates from the concrete near the measuring points, and the steel plates at the web surfaces of the specimen steel plates appeared out-of-plane slippage. It was also found that the load had dropped to 3270 kN (0.71*N*_u_) when the steel tube at measuring point 5 yielded, at which time the specimen was already damaged, and the steel tube at the concave corner still did not provide the confinement effect on the concrete. This further illustrates the poor synergistic working performance of the conventional unstiffened specimen throughout the entire process, from axial loading initiation to destruction, and the weak confinement effect of the steel tube on the concrete.

In Figure 12, when specimen CR3-1 with steel bar truss stiffening was loaded to 4445 kN (0.90 *N*_u_), the steel plate at measuring point 5 yielded. A short compressive stress state then occurred as the load continued, mainly due to the lateral deformation of the concrete of the adjacent column limbs, which squeezed the steel plate at the concave corner. However, as the lateral deformation of the concrete at the distal end of the column limb increased, the steel plate at the web gradually showed a tensile stress state, reaching 175 MPa when the load approached its peak and exceeded the longitudinal compressive stress value at the measuring point. The lateral tensile stress of the steel tube at the concave corner reached 308 MPa as the loading continued until the specimen was damaged.

At measuring point 6, located at 1/2 of the side plate, there was no out-of-plane restriction, resulting in the slow development of lateral stress. The lateral tensile stress at measuring point 6 developed gradually only when the specimen came close to failure.

Therefore, the comparison of stress curves at various locations of the measuring points of both types of specimens reveals that using a steel bar truss stiffening at the concave corners enhances the confinement effect of the steel tube on the core concrete.

**Figure 13:**
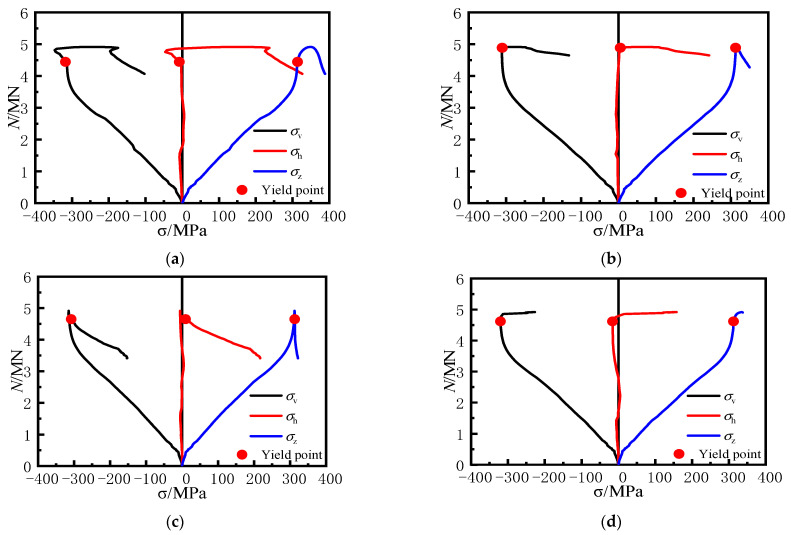
Load–stress curves of the steel tube at the concave corner of specimen CR3-1. (**a**) 5#, (**b**) 8#, (**c**) 11#, (**d**) 14#.

To investigate the impact of steel truss bar stiffening on steel tubes at various positions of the steel tubes, specimen CR3-1 (*s* = 140 mm) is taken as an example. The stress curves of four points at the concave corner of the steel tube were compared and analyzed (Figure 13). The specific arrangement of these points is shown in Figure 4b. Points 5 and 14 are located outside of the steel plate at the weld point and experience direct forces from the diagonal reinforcement, making them strong restraint points. On the other hand, points 8 and 11, situated between the weld points, are not subjected to direct forces from the diagonal reinforcement in the plane where the bending steel is located, making them weak restraint points. Before the peak load was reached, the steel plates at points 5 and 14 had already yielded. Upon reaching the peak load, the lateral tensile stress in the steel tube increased significantly, and the steel plates at these two points were better restrained by the steel bars. Likewise, the lateral stresses in the steel plates at points 8 and 11 also increased significantly after reaching the peak load, indicating that the steel plates were still well restrained by the steel bars at the weak restraint points. In conclusion, for specimen CR3-1, with a node spacing of 140 mm, the setting of steel bar truss stiffening has a substantial restricting effect on the steel tube at the concave corners of the sections at different heights, which enhances the confinement effect of the steel tube on the core concrete.

**Figure 14:**
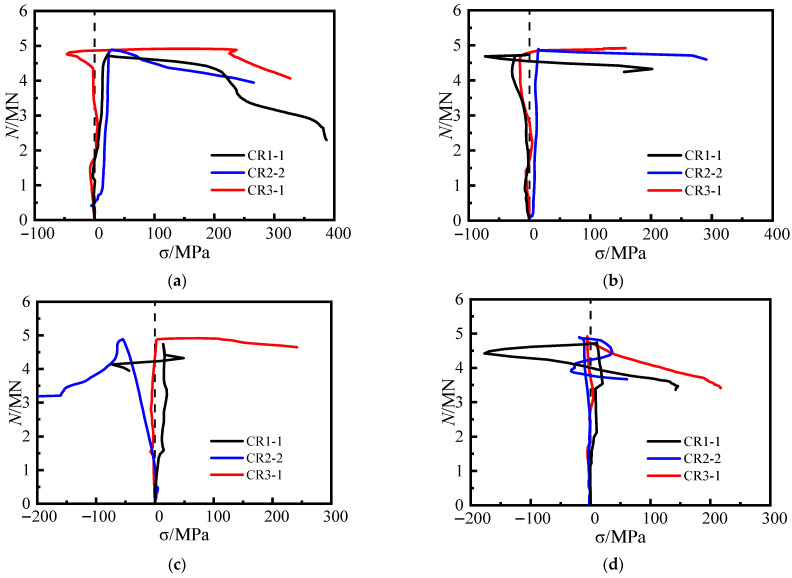
The transverse stress curves of steel tubes at the shaded corner. (**a**) 5#, (**b**) 14#, (**c**) 8#, (**d**) 11#.

Figure 14a shows the lateral load stress curves of the steel tube at measuring point 5 for various specimens, where the node spacing of the steel bar truss is altered. For CR2-2 (*s* = 200 mm) and CR1-1 (*s* = 280 mm) specimens, the lateral stress at measuring point 5 did not increase significantly before reaching the peak load. However, after the peak load, the tensile stress increased rapidly until the specimens were damaged, reaching 209 MPa and 223 MPa, respectively. This indicates that the effect of steel bars on the truss is not noticeable before reaching the peak load as the node spacing increases. Only after the peak load does the rebar play a restrictive role on the steel tube at the concave corner. The lateral load stress curves at measuring point 14 shown in Figure 14b support this conclusion. In contrast, the lateral stress curves at the weakly restrained point 8 or point 11, measured at different node spacings, show more marked differences (Figure 14c,d).

**Figure 15:**
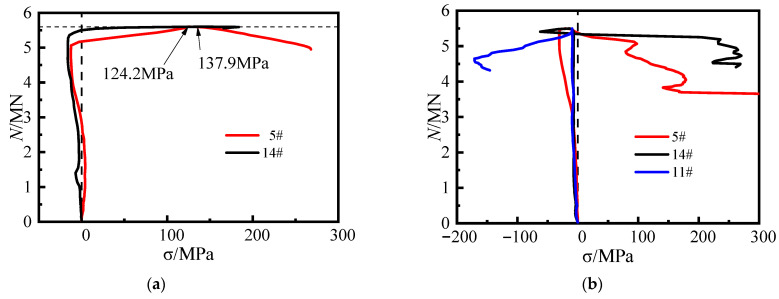
Transverse stress curve of steel tube. (**a**) CR5-1, (**b**) CR4-1.

Specimen CR5-1 (*α*_1_ = 13.17%; *s* = 140 mm), shown in Figure 15a, exhibited a similar pattern to specimen CR3-1 (*α*_1_ = 8.24%; *s* = 140 mm) at both points 5 and 14. At the peak load, the lateral tensile stresses in the steel tube at points 5 and 14 of the specimen were 124.2 MPa and 137.9 MPa, respectively. The lateral tensile stress of the steel tube continued to increase rapidly after reaching the peak load. The development of the lateral stress curve of the steel plate at the concave corner in Figure 15b of specimen CR4-1 (*α*_1_ = 13.17%; *s* = 200 mm) is generally consistent with that of specimen CR2– 2 (*α*_1_ = 8.24%; *s* = 200 mm). This indicates that when the steel ratio of the steel tube is increased, the steel bars in the truss continue to effectively restrict the out-of-plane deformation of the steel plates at the concave corners.

In summary, the steel tubes at the concave corners of the cross-shaped CFST stub column with steel bar truss stiffening can effectively restrain the lateral expansion of the core concrete and enhance the synergistic working effect between the steel tube and the core concrete. When the steel bar truss node spacing is reduced to 140 mm, the steel tube at the concave corner can provide effective confinement to the core concrete.

### 4.3. Longitudinal Stress of Concrete

**Figure 16:**
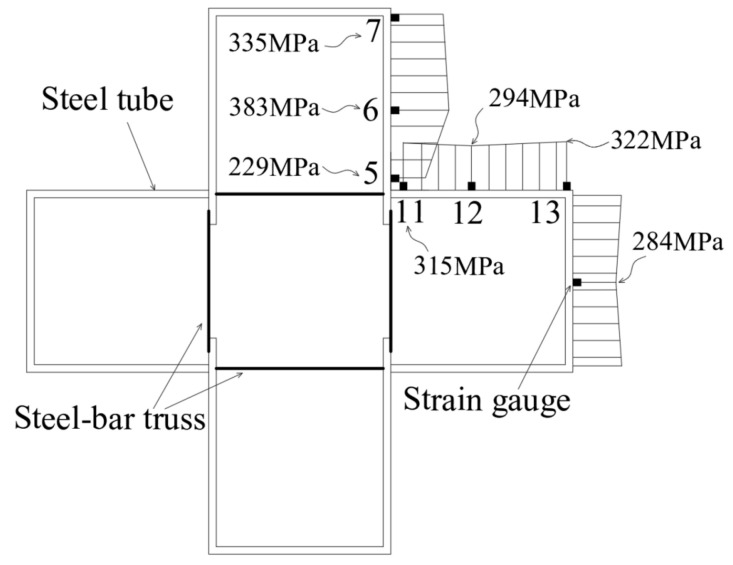
Diagram of longitudinal stress of steel tubes of CR3-1 under peak load.

Based on the failure patterns of typical specimens (Figure 5), the mid-height of the specimen is considered to be the damaged section of the specimen. Figure 16 illustrates a schematic of the longitudinal stress distribution of the steel tube in the damaged section of specimen CR3-1 under peak load, and the longitudinal stress in the steel tube between the measuring points is obtained through the linear difference method. Furthermore, based on the superposition principle, the longitudinal stresses of the steel tube and steel bars at the damaged section are utilized to calculate the average longitudinal stress of concrete against the axial deformation during the test. During the calculation process, it is assumed that the longitudinal stress of the concrete is uniformly distributed on the cross-section.

**Figure 17:**
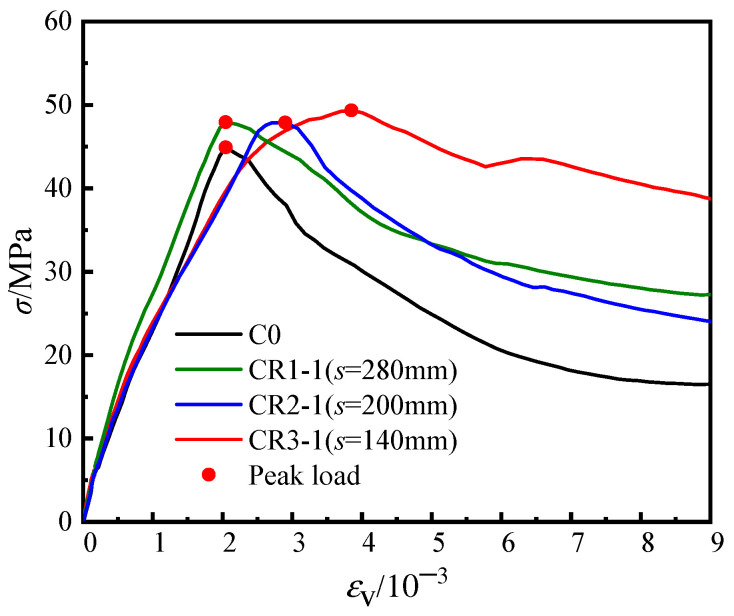
Stress-averaged longitudinal strain curve of core concrete.

The result against the axial deformation of the specimens is given in Figure 17. By comparing the curves, the development of the average longitudinal stress *σ*-average longitudinal strain *ε*_v_ curve of the concrete in each type of cross-section specimen was basically the same at the initial stage of loading, indicating that steel bar truss stiffening had little effect on the initial stiffness of the core concrete. When the specimens entered the elastic–plastic stage, for unstiffened specimen C0, the plastic development of the core concrete was not evident before the peak load due to the inability of the external steel tube to confine the core concrete in time after the concrete had cracked. When the steel bars were set up inside the member, the confinement effect of the steel tube on the concrete was enhanced, delaying the extension of concrete cracks and improving the compressive strength and axial deformation capacity of the core concrete. Before the peak load, the curve for specimen CR1-1 with a steel bar truss node spacing of 280 mm was similar to that of specimen C0, with the curve basically showing a linear development trend and the confinement effect of the steel tube on the core concrete was not significantly enhanced. In contrast, for specimen CR2-1 (*s* = 200 mm) and specimen CR3-1 (*s* = 140 mm), the curves develop non-linearly, and the axial deformation capacity is significantly enhanced. Additionally, the smaller the steel bar truss node spacing, the stronger the axial deformation capacity of the concrete. When the load reached the peak, the ultimate compressive strength of the core concrete of CR1-1, CR2-1, and CR3-1 was 6.8%, 6.6%, and 9.7% higher than that of C0, respectively. Meanwhile, after loading to the peak load, the concrete longitudinal stress curves of the specimens with steel bar truss stiffening decreased at a slower rate compared to the unstiffened specimen and maintained a higher value when the specimen was damaged. Comparing the curves of the specimens with steel bar truss stiffening, it is also found that the smaller the steel bar truss node spacing, the smoother the curve decreases. It shows that steel bar truss stiffening can improve the compressive strength of the core concrete to a certain extent and can also effectively improve the deformation capacity of the core concrete, and the denser the steel bar truss arrangement, the more obvious the effect.

**Figure 18:**
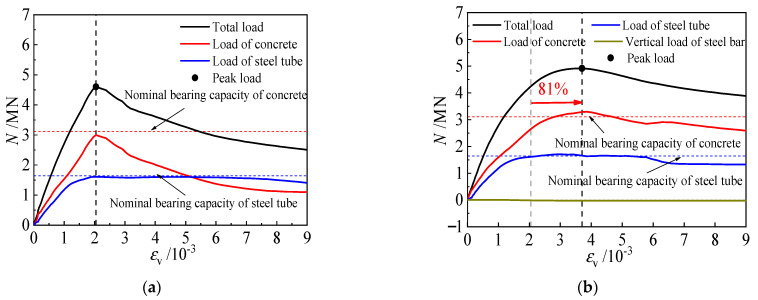
Load-strain curve of each component of specimens. (**a**) C0, (**b**) CR3-1.

Figure 18 presents the load-averaged longitudinal strain curves for each component of typical specimens. The load development of the steel tubes is comparable, with both reaching approximately 2000 micro-strains when the load on the tube remained constant. The mechanical behavior of the concrete significantly impacts the bearing capacity and ductility of the member, as demonstrated by the superposition principle. In specimen CR3-1, the longitudinal strain increases by 81% when the peak load is reached, in contrast to unstiffened specimen C0. Moreover, the full process comparison curve of the concrete indicates that the axial deformation capacity of the concrete is improved by steel bar truss stiffening, which, in turn, increases the ductility of the cross-shaped CFST stub column to a greater extent.

The compressive strength of the concrete was compared to the average longitudinal stress (Table 4). For unstiffened specimen C0, the average longitudinal stress of concrete was found to be 3.7% lower. However, for specimens CR1-1 (*α*_1_ = 8.24%; *s* = 280 mm), CR2-1 (*α*_1_ = 8.24%; *s* = 200 mm), and CR3-1 (*α*_1_ = 8.24%; *s* = 140 mm) with steel bar truss stiffening, the average longitudinal stress of concrete was higher than the compressive strength by 2.9%, 2.6%, and 5.7%, respectively. Increasing the steel ratio of the steel tube to 13.17% resulted in a 3.2% and 6.2% increase in percentage for specimens CR4-1 (*α*_1_ = 13.17%; *s* = 140 mm) and CR5-1 (*α*_1_ = 13.17%; *s* = 200 mm), respectively. These results suggest that the setting of steel bar truss stiffening can improve the compressive strength of concrete to a certain extent. The compressive strength can be increased by approximately 6% when the steel bar truss nodal spacing is reduced to 140 mm.

In summary, the steel bars welded to the inner side of the steel tubes at concave corners can enhance the confinement effect of the steel tube on the concrete. Moreover, an appropriate arrangement of steel bar truss node spacing can significantly increase the deformation capacity of the core concrete. Additionally, it has the potential to improve the compressive strength of the concrete to a certain extent.

## 5. Calculation Method of Bearing Capacity

In order to study the calculation method of the bearing capacity of the cross-shaped CFST stub column, the test results were analyzed and compared with the domestic and foreign CFST structural design codes.

At present, there is no relevant formula concerning the bearing capacity of cross-sectional steel tubes and concrete members in foreign countries; therefore, the design method of using a CFST with a square section is used for the calculation, the foreign codes of this kind mainly have European code EC4 (2004) [27], American steel design code ANSI/AISC 360-10 [28], and British code BS5400 [29].

The relevant calculation formulas are as follows:

(4) $$N_{\mathrm{EC}4}=A_{s}f_{y}+A_{c}{f^{\prime }}_{c}$$

(5) $$N_{\mathrm{AISC}}=A_{s}f_{y}+0.85A_{c}{f^{\prime }}_{c}$$

(6) $$N_{\mathrm{BS}}=A_{s}f_{y}+0.675A_{c}f_{\mathrm{cu}}$$

where *N*_Ec4_, *N*_AISC_, and *N*_bs_ are the compressive strength of the CFST columns, *f*_y_ is the yield strength of the steel, *f*′ is the compressive strength of the concrete cylinder, *f*_cu_ is the cube compressive strength, *A*_s_ is the section area of the steel tube, and *A*_c_ is the section area of the concrete.

For the study of the bearing capacity calculation method of the cross-section and square steel tube concrete members, there have been several codes in China, and the relevant codes are mainly GB 50936-2014 [30], DBJ 13-51-2003 [31], and CECS159-2018 [32], and the relevant calculation equations are shown below. The influence of the restraining effect of the steel tube on the concrete is considered in the first two calculation equations.

(7) $$N_{\mathrm{GB}}=A_{\mathrm{sc}}(1.212+B\xi +C\xi^{2})f_{c}$$

(8) $$N_{\mathrm{BDJ}}=A_{\mathrm{sc}}(1.18+0.85\xi )f_{c}$$

(9) $$N_{\mathrm{CECS}}=A_{s}f_{y}+A_{c}f_{c}$$

where *A*_sc_ is the combined cross-sectional area, *A*_sc_ = *A*_s_ + *A*_c_, *ξ* is the constraint effect coefficient, *ξ* = *A*_s_*f*_y_/(*A*_s_ *f*_c_), *B* and *C* are calculated coefficients, *B* = 0.131*f*/213 + 0.723, *C* = − 0.070*f*_c_/14. 4 + 0.026.

A comparison in terms of the bearing capacity of the calculation results in the different design specifications and the test results are given in Table 5. The results reveal that the design values of GB50936-2014 and DBJ13-51-2003 are larger than the test values and are on the unsafe side. The main reason is that these two methods overestimate the confinement effect of steel tubes on concrete. The steel bar truss stiffening in cross-shaped CFST columns can increase the confinement to a limited extent, but the confinement effect of steel tubes on concrete is still smaller than that in square CFST columns due to irregular cross-sectional forms; therefore, these two methods are not suitable. Additionally, the calculation results of ANSI/AISC 360-10 and BS5400 are smaller than the test values and show a much safer prediction. In contrast, the results obtained in terms of EC4 (2004) and CECS 159-2018 are in good agreement with the test values, and these two calculation methods give the mean values of 0.99 and 0.97 with the coefficient of variation of 0.035 and 0.034, respectively, which shows that they have high accuracy and stability in the prediction of the compressive strength of the cross-shaped CFST stub columns with steel bar truss stiffening. Therefore, within the parameters of this paper, the calculation methods in EC4 (2004) and CECS159-2018 are recommended to be employed for the calculation in the bearing capacity of the cross-shaped concrete-filled steel tubular stub column with steel bar truss stiffening.

## 6. Conclusions

In this paper, seven types of tests on cross-shaped CFST stub columns under axial compression have been described. Based on the test results, the following conclusions can be drawn within the scope of this study.

1. Due to the irregular cross-sections of the conventional cross-shaped CFST stub columns, the confinement effect of the steel tube on the concrete is insufficient under axial compressive load. With the steel bars in the truss employed, the deformation mode of the steel plate was changed from single-wave buckling to multiple-wave buckling, and the core concrete also tended toward multiple-section crushing failure.
2. Despite employing a 0.5% steel ratio for the steel bars, the steel bars, which were welded to the inside of the steel tube at the adjacent concave corners, increased the confinement of the tube to the concrete, thereby improving the bearing capacity and ductility of the member. In comparison to the unstiffened specimen, the bearing capacity and ductility of specimen CR3-1 increased by 6.8% and 93%, respectively, with a significantly improved ductility coefficient of 4.4.
3. In the cross-shaped CFST stub column with steel bar truss stiffening, steel bar truss node spacing plays a significant role in the restraining effect on the steel tube. Only when the node spacing is reduced to a certain value can the steel bars exert their tensile forces on the steel tube at the concave corners, thereby enhancing the confinement effect of the steel tube on the concrete. For specimen CR3-1, with a steel bar truss node spacing of 140 mm, the steel bars begin to restrict the lateral deformation as the specimen enters the elastic–plastic stage. Compared with specimens CR2-1 (*s* = 200 mm) and CR1-1 (*s* = 280 mm), specimen CR3-1 shows better ductility, with its ductility coefficient increasing by 57.1% and 51.7%, respectively. This indicates that for the parameters used in this paper when the steel bar truss node spacing is reduced to 140 mm, the ductility of the cross-shaped CFST stub column with steel bar truss stiffening can be significantly improved.
4. Upon comparing the design code methods with the test results, both EC4 (2004) and CECS 159-2018 are in good agreement with the test results. The former provides a safe prediction within 1%, and the latter provides a safe prediction within 3%. Therefore, based on the parameters in this paper, Eurocode 4 (2004) and Chinese code CECS159-2018 are recommended for the prediction of the axial bearing capacity of cross-shaped CFST stub columns with steel bar truss stiffening.

## Figures and Tables

**Table 1:** Main parameters and test results.

Specimen	*t* (mm)	*s* (mm)	*α*_1_ (%)	*α*_2_ (%)	*α* (%)	*N*_0_ (kN)	*N*_u_ (kN)	*SI*	*ε*_0.75_ (10^−3^)	*ε*_0.85_ (10^−3^)	*DI*
C0	3.50	—	8.24	—	8.24	4752	4604	0.97	1359	3141	2.31
CR1-1	3.50	280	8.24	0.42	8.66	4752	4740	1.00	1330	3810	2.86
CR1-2	3.50	280	8.24	0.42	8.66	4752	4646	0.98	1289	3802	2.95
CR2-1	3.50	200	8.24	0.49	8.73	4752	4783	1.01	1534	4479	2.92
CR2-2	3.50	200	8.24	0.49	8.73	4752	4893	1.03	1477	3995	2.70
CR3-1	3.50	140	8.24	0.57	8.81	4752	4917	1.03	1569	6921	4.41
CR3-2	3.50	140	8.24	0.57	8.81	4752	4755	1.00	1569	6900	4.40
CR4-1	5.35	200	13.17	0.54	13.71	5210	5498	1.06	1400	5245	3.75
CR4-2	5.35	200	13.17	0.54	13.71	5210	5597	1.07	1424	4922	3.46
CR5-1	5.35	140	13.17	0.62	13.79	5210	5602	1.08	1433	8646	6.03
CR5-2	5.35	140	13.17	0.62	13.79	5210	5543	1.06	1634	9612	5.88
CM6	3.50	—	10.29	—	10.29	5548	6000	1.08	1939	7754	4.00

Note: The specimens are numbered according to the following rules. The first letter, C, is the abbreviation of column, and the second letter, R or M, is the abbreviation of rebar or multicavity, respectively. The first number behind the letter represents the group, and the second number represents the serial number of the specimen in the corresponding group.

**Table 2:** Mechanical properties of steel.

Steel	*f*_y_ (MPa)	*f*_u_ (MPa)	*E*_s_ (GPa)	*μ*_s_
Steel plate (3.50 mm)	313.1	455.5	205	0.282
Steel plate (5.35 mm)	278.0	441.9	195	0.284
Steel bar (8 mm)	539.4	600.0	203	0.291

**Table 3:** Stress table of steel bars for specimens.

Specimen	*σ*_c, max_ (MPa)	*N*_σ0/*N*u_	*σ*_t, *N*u_ (MPa)	*σ*_t, *N*0.85_ (MPa)
CR1-1	83.7	1	0	459.1
CR2-1	37.4	0.92	396.3	577.3
CR3-1	8.9	0.65	320.8	492.7
CR4-1	72.8	0.98	41.9	520.0
CR5-1	12.4	0.86	164.3	570.7

Note: *σ*_c, max_ is the maximum compressive stress of the steel bar. *N*_σ0/*N*u_ is the normalized load when the stress in the reinforcement is zero. *σ*_t, *N*u_ is the tensile stress of the steel bar at peak load. *σ*_t, *N*0.85_ is the tensile stress in the steel bar when it drops to 85% of the peak load.

**Table 4:** Comparison table of strength of the core concrete of the specimens.

Specimens	The Compressive Strength *f*_c_ (MPa)	The Average Longitudinal Stress *σ* (MPa)	Increase in Percentage
C0	46.6	44.9	−3.7%
CR1-1	46.6	47.9	2.9%
CR2-1	46.6	47.8	2.6%
CR3-1	46.6	49.2	5.7%
CR4-1	46.6	48.1	3.2%
CR5-1	46.6	49.5	6.2%

**Table 5:** Comparison between the test results and the calculation results of the relevant codes.

Specimen	*T* (mm)	*S* (mm)	*N*_u_ (kN)	*N*_EC4_/*N*_u_	*N*_AISC_/*N*_u_	*N*_BS_/*N*_u_	*N*_GB_/*N*_u_	*N*_DBJ_/*N*_u_	*N*_CECS_/*N*_u_
CR1-1	3.5	280	4740	1.03	0.92	0.93	1.10	1.15	1.00
CR1-2	3.5	280	4646	1.05	0.94	0.95	1.12	1.18	1.02
CR2-1	3.5	200	4783	1.02	0.92	0.92	1.09	1.14	0.99
CR2-2	3.5	200	4893	0.99	0.89	0.90	1.06	1.12	0.97
CR3-1	3.5	140	4917	0.99	0.89	0.89	1.06	1.11	0.97
CR3-2	3.5	140	4755	1.02	0.92	0.92	1.10	1.15	1.00
CR4-1	5.35	200	5498	0.97	0.88	0.94	1.01	1.11	0.95
CR4-2	5.35	200	5597	0.95	0.87	0.92	1.00	1.09	0.93
CR5-1	5.35	140	5602	0.95	0.87	0.92	1.00	1.09	0.93
CR5-2	5.35	140	5543	0.96	0.87	0.93	1.01	1.10	0.94
Mean value				0.99	0.90	0.92	1.05	1.12	0.97
COV (Coefficient of variation)				0.035	0.029	0.017	0.045	0.027	0.034

## Data Availability

All data included in this study are available upon request by contacting the corresponding author.
